# Improved osteogenesis and upregulated immunogenicity in human placenta-derived mesenchymal stem cells primed with osteogenic induction medium

**DOI:** 10.1186/s13287-016-0400-6

**Published:** 2016-09-20

**Authors:** Xuejie Fu, Huilin Yang, Hui Zhang, Guichao Wang, Ke Liu, Qiaoli Gu, Yunxia Tao, Guangcun Chen, Xiaohua Jiang, Gang Li, Yanzheng Gu, Qin Shi

**Affiliations:** 1 Orthopedic Department, The First Affiliated Hospital of Soochow University, Orthopedic Institute, Soochow University, Suzhou, 215006 People’s Republic of China; 2 Key Laboratory of Nano-Bio Interface, Division of Nanobiomedicine and i-Lab, Suzhou Institute of Nano-Tech and Nano-Bionics, Chinese Academy of Sciences, Suzhou, 215123 People’s Republic of China; 3 Epithelial Cell Biology Research Center, School of Biomedical Sciences, Faculty of Medicine, The Chinese University of Hong Kong, Hong Kong, SAR People’s Republic of China; 4 Department of Orthopaedics & Traumatology, Stem Cells and Regenerative Medicine Laboratory, Li Ka Shing Institute of Health Sciences, The Chinese University of Hong Kong, Hong Kong, SAR China; 5 Key Laboratory of Stem Cell of Jiangsu Province, Institute of Medical Biotechnology, Soochow University, No.188 Shizi Street, Suzhou, 215006 People’s Republic of China

**Keywords:** Mesenchymal stem cells, Dedifferentiated mesenchymal stem cells, Osteogenesis, Immunogenicity

## Abstract

**Background:**

Mesenchymal stem cells (MSCs) are widely used in cell-based therapy owing to their multilineage potential and low immunogenicity. However, low differentiation efficiency and unpredictable immunogenicity of allogeneic MSCs in vivo limit their success in therapeutic treatment. Herein, we evaluated the differentiation potential and immunogenicity of human placenta-derived MSCs manipulated with osteogenic priming and dedifferentiation process.

**Methods:**

MSCs from human placentas were subjected to osteogenic induction and then cultivated in osteogenic factor-free media; the obtained cell population was termed dedifferentiated mesenchymal stem cells (De-MSCs). De-MSCs were induced into osteo-, chondro- and adipo-differentiation in vitro. Cell proliferation was quantified by a Cell-Counting Kit-8 or tritiated thymidine ([^3^H]-TdR) incorporation. Meanwhile, the osteogenesis of De-MSCs in vivo was assayed by real-time PCR and histological staining. The expressions of stem cell markers and co-stimulatory molecules on De-MSCs and lymphocytes from primed BALB/c mouse with De-MSCs were determined by flow cytometry.

**Results:**

De-MSCs exhibited some properties similar to MSCs including multiple differentiation potential and hypoimmunogenicity. Upon re-osteogenic induction, De-MSCs exhibited higher differentiation capability than MSCs both in vitro and in vivo. Of note, De-MSCs had upregulated immunogenicity in association with their osteogenesis, reflected by the alternated expressions of co-stimulatory molecules on the surface and decreased suppression on T cell activation. Functionally, De-MSC-derived osteoblasts could prime lymphocytes of peripheral blood and spleen in BALB/c mice in vivo.

**Conclusions:**

These data are of great significance for the potential application of De-MSCs as an alternative resource for regenerative medicine and tissue engineering. In order to avoid being rejected by the host during allogeneic De-MSC therapy, we suggest that immune intervention should be considered to boost the immune acceptance and integration because of the upregulated immunogenicity of De-MSCs with redifferentiation in clinical applications.

## Background

Mesenchymal stem cells (MSCs) isolated from many tissues are fibroblast-like adult stem cells, characterized by the potential of self-renewal and multilineage differentiation [1]. In addition, MSCs can suppress or ameliorate immune responses by regulating the expression of co-stimulatory molecules on the surface of themselves, secreting soluble negative cytokines and modulating the activity of major immune cell populations, such as dendritic cells (DC), T cells, B cells and natural killer (NK) cells [2, 3], and inducing immune cell division arrest and apoptosis. Therefore, the applications of MSCs have been widely studied in animal models and in human for treating autoimmune diseases and tissue engineering [4–6].

MSCs are considered to be immunoprivileged because of their low immunogenicity, but a large body of evidence has demonstrated that differentiated MSCs could initiate immune response and impair cell-based therapy, especially allogeneic (allo)-MSC-based therapy [7–10]. Huang et al. demonstrated that MSCs acquired endothelial, myogenic or smooth muscle characteristics, in association with enhancing the expression of major histocompatibility complex (MHC)-Ia and -II (immunogenic), reducing the expression of major histocompatibility complex-Ib (immunosuppressive) and promoting cytotoxicity in co-culture with allogeneic leukocytes. When allogeneic MSCs were administered into the infracted myocardium after a myocardial infarction in rats, cells were eliminated from the heart by 5 weeks after implantation, and their therapeutic benefits were lost within 5 months. Consistent with in vitro data, these cells expressed the differentiated markers and high levels of MHC-Ia and MHC-II after implantation [11]. Lohan et al further summarized that during osteogenic, chondrogenic and myocardial differentiation, the expressions of MHC-I, MHC-II, CD80 or CD86 on differentiated allo-MSCs increased, while the secretion of prostaglandin E2 (PGE2) or nitric oxide (NO) reduced [12]. It is indicative of the effect of induced differentiation on the decreased immunosuppressive ability, and increased immunogenicity of allo-MSCs is a potential obstacle when applying MSCs in tissue replacement therapies. Therefore, the immunogenicity of differentiated MSCs needs to be fully considered.

Though MSCs are the potential regenerative cells in tissue engineering, low in vivo survival and differentiation potential of transplanted MSCs are detrimental to their overall effectiveness and thus clinical usage [6, 13]. Thus, it is of great significance to search for alternative seed cells which have hypoimmunogenicity, longer survival, and higher differentiated efficiency in MSC-based therapy.

Dedifferentiation has been considered as one of the mechanisms rerouting cell fate by reverting differentiated cells to an earlier, more primitive phenotype characterized by alternating gene expression patterns which confer an extended differentiation potential [14]. Previously, it was reported that MSC-derived neurons, upon withdrawal of extrinsic neuronal induction, could revert to the original MSC-like state. The obtained cell population was termed dedifferentiated MSCs (De-MSCs), which expressed markers of MSCs and could be redifferentiated into neurons or transdifferentiated into epithelial cells [15–17]. Interestingly, compared to the undifferentiated MSCs, De-MSCs redifferentiated into neurons more efficiently and survived longer in unfavorable environments [15, 17]. Similarly, upon withdrawal of extrinsic factors, chondrocytes were able to revert back to MSCs morphologically and had improved proliferative and differential potential [15, 18, 19]. These studies on De-MSCs, related to the characteristics of dedifferentiated cells, provided new insights for stem cell transplantation. For the ultimate application of De-MSCs, the characteristics of De-MSCs remain to be addressed; furthermore the alteration of immunogenicity during redifferentiation needs to be explored.

In the present work, we prepared MSCs from human placentas. Meanwhile, we established the cell population of De-MSCs by adding and withdrawing osteoblast-inducible factors in sequence. We further identified that De-MSCs had multidifferentiation potential and shared some phenotypic characteristics similar to MSCs. Aiming for the possibility of allogeneic De-MSCs application in clinic, we first compared the osteogenic potential of MSCs and De-MSCs, and then we explored the impact of osteogenic differentiation on the immunogenicity of MSCs and De-MSCs.

## Methods

Under the approval of the Ethics Committee of the First Affiliated Hospital of Soochow University, human placentas and peripheral blood samples were obtained after healthy donors gave written informed consent.

The animal studies were performed following the Guide for the Care and Use of Laboratory Animals of the US National Institutes of Health (NIH), and licensed by the Animal Research Committee of the First Affiliated Hospital of Soochow University.

### Preparation of MSCs, Ob-MSCs, De-MSCs and Re-MSCs

Normal full-term human placentas (≥37 weeks gestational age) were collected from healthy donors. Placenta MSCs were isolated and cultured as described previously [20]. The tissue was digested with 0.01 % collagenase type II solution, and then filtered through a 100-μm cell strainer. The cells were centrifuged and resuspended in Dulbecco’s modified Eagle’s medium-low glucose (DMEM-LG, Hyclone, Logan, UT, USA) supplemented with 10 % fetal bovine serum (FBS, Hyclone, USA), 100U/mL penicillin G, 100 μg/mL streptomycin, and then seeded in 6-well plates. Cells were incubated at 37 °C in a 5 % CO_2_ atmosphere. Nonadherent cells were removed after 3 days, and the medium was replaced every 3 days. When cells reached approximately 70–80 % confluence, the adherent cells were trypsinized by 0.05 % trypsin/EDTA and expanded.

To obtain De-MSCs, MSCs were cultured in osteogenic induction medium for 7 days and then subjected to complete medium (DMEM-LG with 10 % FBS) without inducible factors for 2 weeks. The osteogenic induction medium contains 0.1 μM dexamethasone (Sigma-Aldrich, St. Louis, MO, USA), 10 mM β-glycerolphosphate (Sigma-Aldrich, USA) and 0.25 mM ascorbate (Sigma-Aldrich, USA) in DMEM-HG (Hyclone, USA) with 10 % FBS. To gain MSC-derived osteoblasts (Ob-MSCs), MSCs were subjected to osteogenic induction medium for 7 days. Similarly, after osteogenic induction of De-MSCs for 7 days, osteoblasts derived from De-MSCs (Re-MSCs) were obtained.

### Multilineage differentiation of MSCs and De-MSCs

To induce osteoblasts, both MSCs and De-MSCs were subjected to osteogenic medium for 21 days. Then, cells were incubated for 30 mins with Alizarin Red S (Sigma-Aldrich, USA) at pH 4.1 at room temperature to evaluate calcium accumulation.

For adipogenesis, cells were seeded in 6-well plates and treated in DMEM-HG (Hyclone, USA) with 1 μM dexamethasone (Sigma-Aldrich, USA), 10 μg/mL insulin (Sigma-Aldrich, USA), 0.5 mM 3-isobutyl-1-methylxanthine (IBMX, Sigma-Aldrich, USA), 0.2 mM indomethacin (Sigma-Aldrich, USA) and 10 % FBS. The adipogenic differentiation medium was changed every 3 days for 2 weeks. Oil Red O (Sigma-Aldrich, USA) was used to visualize lipid-rich vacuoles.

For chondrogenic differentiation, approximately 2–3 × 10^6^ cells in 0.5 mL medium were centrifuged at 500 rpm/min for 10mins in a 15 mL polypropylene tube to form a pellet. Without disturbing the pellet, cells were cultured for 21 days in DMEM-HG supplemented with 10 % FBS, 10 ng/mL transforming growth factor beta 1 (TGF-β1) (Peprotech, Rocky Hill, NJ, USA), 0.5 μg/mL of insulin (Sigma-Aldrich, USA), 50 μM ascorbic acid (Sigma-Aldrich, USA). On day 21, cells were kept in 10 % formalin for 1 h at room temperature, dehydrated in serial ethanol dilutions, and embedded in paraffin blocks. Paraffin sections were stained histologically with Toluidine Blue solution (Sigma-Aldrich, USA) to demonstrate the presence of intracellular matrix mucopolysaccharides.

### Cell proliferation evaluation and alkaline phosphatase (ALP) assay of MSCs and De-MSCs osteogenesis in vitro

The viability and proliferation of MSCs and De-MSCs were measured in osteogenic medium with a Cell-Counting Kit-8 (CCK-8, Dojindo, Kumamoto, Japan) for 7 days. At the desired time points, cells were incubated in CCK-8 solution for 2 h. The absorbance was read at 450 nm by microplate reader (BioTek, Winooski, VT, USA) and the live cell number was correlated to optical density (OD).

MSCs and De-MSCs (10^4^ cells/cm^2^) were seeded in 6-well plate and replaced with osteogenic induction medium. ALP activity was respectively assayed before (0 days) and 7, 14, and 21 days after induction, by a BCIP/NBT alkaline phosphatase color development kit (Beyotime Institute of Biotechnology, Haimen, China) according to the manufacturer’s instructions, and ALP-positive cells were stained in blue.

### Osteogenesis of MSCs and De-MSCs in vivo

Collagen scaffolds were prepared according to the previous method [21] and cut into 3 mm × 3 mm × 3 mm collagen bundles. A total of 50 μL suspension of the MSCs or De-MSCs cells (1 × 10^6^ cells/bundle) was seeded on the collagen scaffolds and then implanted subcutaneously in the back of nonobese diabetic/severe combined immunodeficient mouse recipients (NOD/SCID). Seven days after transplantation, the scaffolds were collected and digested by collagenase to harvest the loaded cells. To investigate the osteogenic capability of MSCs and De-MSCs in vivo, ALP activity assay was performed on some cells, the others were evaluated for expression of human osteogenesis-related genes by quantitative real-time PCR assays (qRT-PCR). Thirty days later, the implants were gathered, fixed and sliced for hematoxylin and eosin (H&E) staining and immunohistochemical staining for collagen II expression (rabbit anti-human collagen II, Abcam, Cambridge, MA, USA).

### qRT-PCR for gene expression

To evaluate the osteogenic differentiation potential of MSCs and De-MSCs, the gene expression at the mRNA level was examined. Seven days after induction in vitro or implantation in vivo, total RNA of different cells were extracted using Trizol reagent (Invitrogen, Waltham, MA, USA) and reverse-transcribed into complementary DNA (cDNA) with the M-MLV reverse transcriptase kit (TaKaRa, Shiga, Japan). By using a Brilliant SYBR Green QPCR Master Mix (TakaRa, Japan), the cDNA templates were subjected to qRT-PCR to semi-quantify the gene expressions of human bone morphogenetic protein 2 (BMP2), human Runt-related transcription factor 2 (Runx2) and human Osterix (Osx), normalized to the expression of human glyceraldehyde-3-phosphate dehydrogenase (GADPH). BMP2: forward primer 5′-GCACCAAGATGAACACAG-3′, reverse primer 5′-AGGGCATTCTCCGTGGCAGT-3′; Runx2: forward primer, 5′-TCTTCACAAATCCTCCCC-3′, reverse primer, 5′-TGGATTAAAAGGACTTGG-3′; Osx: forward primer, 5′-CAACTGGCTCTTCTGCGGCAAGAG-3′, reverse primer 5′-GCTGGTGTTTGCTCAGGTGGTC-3′; GAPDH: forward primer, 5′-AGAAGGCTGGGGCTCATTTG-3′, reverse primer 5′-AGGGGCCATCCACAGTCTTC-3′. The relative expression level of target gene was calculated by the 2^−ΔΔCt^ method. Fold was used to show the change times of mRNA level normalized by the undifferentiated MSCs in vitro and differentiated MSCs in vivo.

### Flow cytometry (FCM) analysis for surface markers on cells in vitro

MSCs, Ob-MSCs, De-MSCs and Re-MSCs were collected and FCM analysis was performed with following mouse anti-human monoclonal antibodies (eBioscience, San Diego, CA, USA), FITC-, PE- or APC-conjugated CD29, CD34, CD45, CD90, CD166, CD105, CD28, CD80, CD83, CD86, HLA-DR, MHC-II, programmed cell death 1 ligand 1 (PD-L1), Programmed cell death 2 ligand 1 (PD-L2), B7-homolog 3 (B7-H3, CD276). Conjugated mouse IgG k-chain served as isotype control. Samples were detected by FCM (FC 500FCL, Beckman Coulter, Indianapolis, IN, USA) and analyzed by Flowjo 7.6.1 software (TreeStar, Ashland, OR, USA).

### Mixed lymphocyte reaction (MLR) assay in vitro

Peripheral blood mononuclear cells (PBMCs) from healthy donors were isolated and cultured in RPMI 1640 with 10 % FBS, 100U/mL penicillin G and 100 μg/mL streptomycin. CD3^+^ T cells were isolated from PBMCs using CD3^+^ T cell isolation kit (Life Technologies, Carlsbad, CA, USA) according to the manufacturer’s instructions. MSCs, Ob-MSCs, De-MSCs or Re-MSCs were treated by mitomycin C (MMC, 10 μg/mL, Sigma-Aldrich, USA) for 2 h at 37 °C, then washed three times with PBS containing 1 % FBS.

For MLR assay, T cells (1 × 10^5^ cells per well) with anti-human CD3 and CD28 antibodies (MACS, Miltenyi Biotec, Bergisch Gladbach, Germany) (30 ng/mL, respectively) were cultured in the presence of the MMC-treated MSCs, Ob-MSCs, De-MSCs or Re-MSCs (2 × 10^4^ cells/well). All the T cells were further cultured for 3 days, 1 μCi [^3^H] thymidine was added into the wells 18 hours before the experiment stopped and counts per minute (cpm) was measured by β-scintillation counter (Shimadzu Corporation, Kyoto, Japan).

### Detection of primed lymphocytes by FCM in vivo

Six-week-old female BALB/c mice were immunized with MMC-treated MSCs, Ob-MSCs, De-MSCs and Re-MSCs in normal saline (N.S.), respectively. The mice treated with vehicle were served as control group. 1 × 10^6^ cells in 200 μL N.S. were subcutaneously injected into mice. The PBMCs from mice were harvested 5 days and suspend splenocytes were harvested 7 days after priming. The different cell populations were assayed by FCM. The antibodies specific to mouse (eBioscience, USA) conjugated with PE, FITC, APC were applied to mark mouse CD25, CD4, CD80, CD11b, CD11c, and CD45R (B220).

### Statistical analysis

Values were presented as mean ± standard deviation (SD) and analyzed statistically by GraphPad Prism 5 for Windows (GraphPad Software, San Diego, CA, USA). Two-tailed unpaired Student *t* test was applied between two groups, while one-way ANOVA followed by Tukey’s multiple comparison test was used among more than two groups. Probability values were considered statistically significant at *P* < 0.05.

## Results

### Characterization of MSCs and De-MSCs derived from placentas

**Fig. 1:**
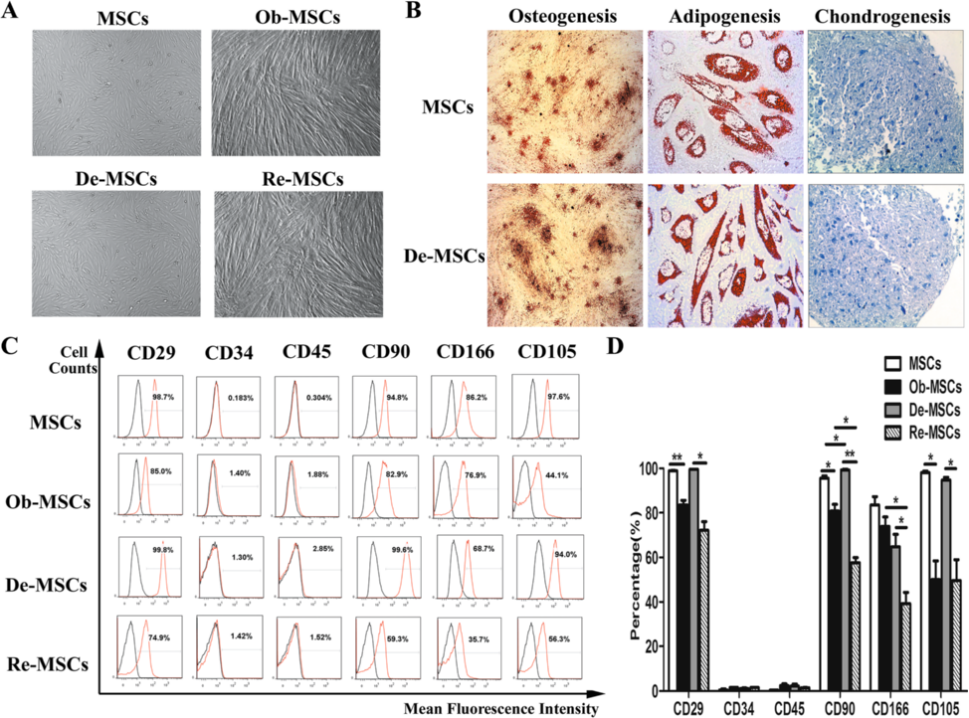
Characterization of human placenta-derived MSCs and De-MSCs. **a** Cell morphology of MSCs (×25), Ob-MSCs (×25), De-MSCs (×25) and Re-MSCs (×25). **b** The staining of Alizarin Red S, Oil Red O and Toluidine Blue respectively for osteogenesis (×25), adipogenesis (×400) and chondrogenesis (×400) of MSCs and De-MSCs. **c** Flow cytometry (FCM) analysis of the expression of indicated cell surface markers in MSCs, Ob-MSCs, De-MSCs and Re-MSCs. **d** Positive rates of FCM presented in the histogram were mean ± SD of three independent experiments. One-way ANOVA test was employed for intergroup comparison. ^*^ *P* < 0.05, ^**^ *P* < 0.01, ^***^ *P* < 0.001. *De-MSCs* dedifferentiated MSCs, *MSCs* mesenchymal stem cells, *Ob-MSCs* osteoblasts differentiated from MSCs, *Re-MSCs* osteoblasts differentiated from De-MSCs

We isolated MSCs from the placentas, which are an important source of MSCs. To identify MSCs and De-MSCs, the cell morphology of MSCs and De-MSCs were first photographed by microscope (Carl Zeiss, Jena, Germany) before and during osteogenesis at different time points. De-MSCs presented fibroblast-like and spindle-shaped morphology, which were similar to MSCs. MSCs-derived osteoblasts (Ob-MSCs) and De-MSCs-derived osteoblasts (Re-MSCs) also had analogous cell morphology. Moreover, Re-MSCs had more calcium nodules than Ob-MSCs, suggesting that De-MSCs have higher osteogenic efficiency than MSCs (Fig. 1a). Both MSCs and De-MSCs possessed the potential to differentiate into osteoblast, adipocytes and chondrocytes, as authenticated by positive staining for Oil red O, Alizarin Red S and Toluidine Blue staining (Fig. 1b), indicating that De-MSCs retained multilineage potential as MSCs. Phenotypic analysis was conducted with MSCs, Ob-MSCs, De-MSCs and Re-MSCs, which were derived from independent placenta samples. Interestingly, De-MSCs were observed to express similar phenotypes when compared with MSCs (Fig. 1c, d). De-MSCs expressed CD29, CD90, CD105, and CD166, the stemness markers of MSCs, but did not express CD34 and CD45 antigen referred to as hematopoietic stem cells. Upon osteogenic induction, Ob-MSCs and Re-MSCs significantly expressed lower CD29, CD90, CD105, and CD166 than those of their counterpart control groups (*P* < 0.05, respectively). Meanwhile, the expressions of CD90 and CD166 on Ob-MSCs were statistically different from those on Re-MSCs (*P* < 0.05, respectively, Fig. 1c, d) Therefore, the data suggest that De-MSCs retain some MSC traits but are distinct from MSCs.

### Enhanced osteogenesis of De-MSCs in vitro

**Fig. 2:**
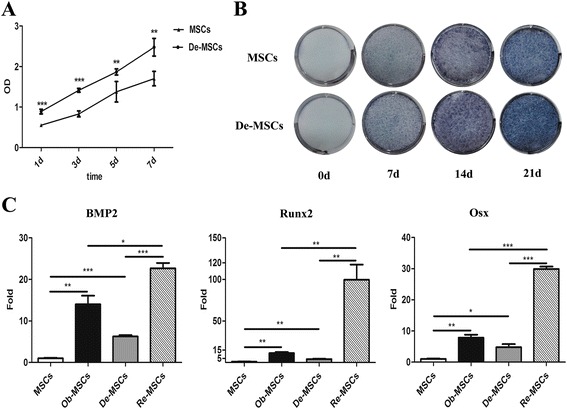
The osteogenic potential of MSCs and De-MSCs in vitro. **a** Proliferation of MSCs and De-MSCs detected by CCK-8 during osteogenesis for 7 days (*n* = 6/group), values of OD presented were mean ± SD, two-tailed unpaired Student *t* test was applied. **b** The ALP staining of MSCs and De-MSCs before (0d) and 7d, 14d, and 21d after osteogenic induction. **c** qRT-PCR analysis of the expression of BMP2, Runx2, Osx (*n* = 3/group), fold values presented as mean ± SD, one-way ANOVA test was employed for intergroup comparison. ^*^ *P* < 0.05, ^**^ *P* < 0.01, ^***^ *P* < 0.001. *ALP* alkaline phosphatase, *BMP2* human bone morphogenetic protein 2, *De-MSCs* dedifferentiated mesenchymal stem cells, *MSCs* mesenchymal stem cells, *Ob-MSCs* MSC-derived osteoblasts, *Osx* Osterix, *Re-MSCs* osteoblasts derived from De-MSCs, *Runx2* human Runt-related transcription factor 2

Upon osteogenic induction, more viable cells were observed in De-MSC group compared to their respective counterparts at the same time point (*P* < 0.01, respectively, Fig. 2a). This could be due to an increase in either cell proliferation or more survival cells. Seven, 14, and 21 days after osteogenic induction, De-MSCs differentiated into osteoblasts vigorously, as illustrated by ALP staining. ALP staining enhanced macroscopically at both early and late stages of differentiated De-MSCs, maybe resulting from the more cells in this group at the early stage and increased ALP activity (Fig. 2b).

After osteogenic induction for 7 days, qRT-PCR was adopted to measure the expression of BMP2, Runx2 and Osx. Compared with the undifferentiated groups, MSCs and De-MSCs, the expressions of BMP2, Runx2 and Osx increased significantly in differentiated groups, Ob-MSCs and Re-MSCs (*P* < 0.01, respectively). Impressively, the expressions of the three genes were statistically higher in De-MSCs than in MSCs (*P* < 0.05, Fig. 2c). Same pattern was observed in Re-MSCs and Ob-MSCs (*P* < 0.05, Fig. 2c). Taken together, the data demonstrated that De-MSCs had higher osteogenic ability compared to MSCs in vitro.

### Promoted osteogenesis of De-MSCs in vivo

**Fig. 3:**
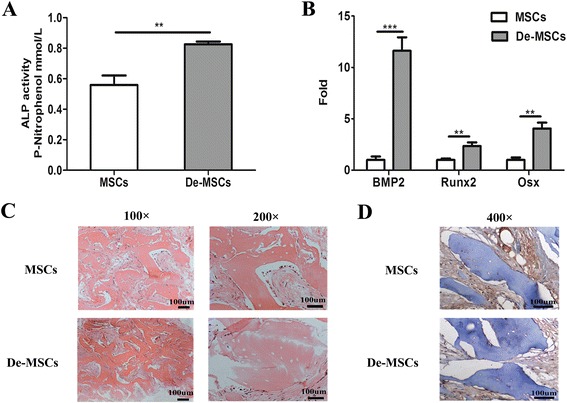
The osteogenic potential of MSCs and De-MSCs in SCID mice. **a**–**d** MSCs or De-MSCs cells (1 × 10^6^ cells) were loaded on the collagen scaffolds and implanted subcutaneously in the back of SCID mice. **a** ALP activity assay of implanted cells 7 days after implantation (*n* = 3/group), values of P-nitrophenol mmol/L presented were mean ± SD; **b** qRT-PCR analysis of implanted cells for the expression of BMP2, Runx2, Osx 7 days after implantation (*n* = 3/group), fold values presented were mean ± SD. **c** The H&E staining for the implanted scaffolds 30 days after implantation. **d** Immunohistological staining of collagen II for the implanted scaffolds 30 days after implantation. ^**^ *P* < 0.01, ^***^ *P* < 0.001. *ALP* alkaline phosphatase, *BMP2* human bone morphogenetic protein 2, *De-MSCs* dedifferentiated mesenchymal stem cells, *MSCs* mesenchymal stem cells, *Osx* Osterix, *Runx2* human Runt-related transcription factor 2

To evaluate osteogenesis in vivo, MSCs and De-MSCs were loaded on collagen scaffolds and transplanted into SCID mouse. The new bone formation was observed in the implants, evidenced by ALP activity, gene expressions of BMP2, Runx2 and Osx and morphological observation. Seven days after transplantation, the ALP activity in the group loaded with De-MSCs was obviously higher than the one with MSCs (*P* < 0.01, Fig. 3a). Compared with the group of MSCs, the mRNA expressions of BMP2, Runx2 and Osx increased significantly in De-MSC group (*P* < 0.01, respectively, Fig. 3b). As expected, the new bone formation and collagen II expression were observed in scaffolds loaded both with MSCs and De-MSCs 30 days after transplantation. The De-MSCs-implanted group had more new bone formation, which further revealed that De-MSCs had a better osteogenic potential compared to MSCs (Fig. 3c, d). This result was consistent with our observations in vitro.

### Upregulated immunogenicity of De-MSCs during osteogenesis

**Fig. 4:**
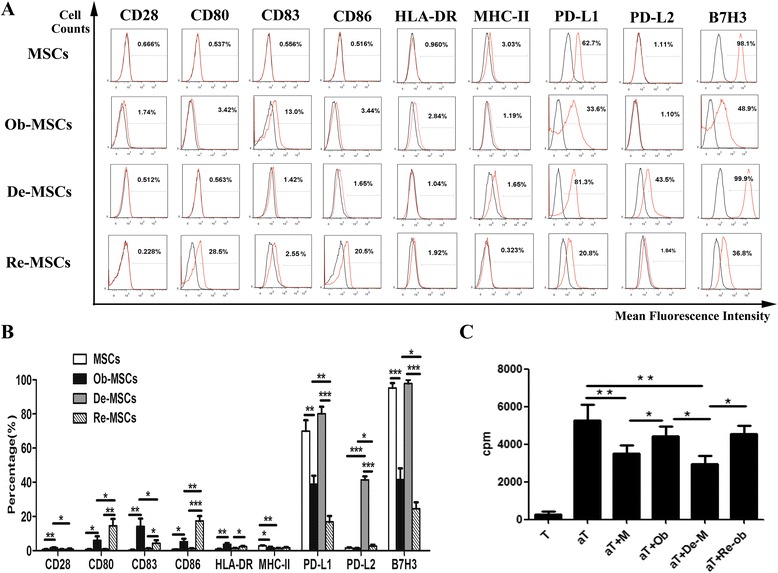
The immunogenic properties of MSCs and De-MSCs in vitro. **a**–**b**Co-stimulatory molecules were detected by FCM. Histograms in *black* showed isotype control staining and histograms in *red* showed the specific expression of the indicated cells. Values of positive rate presented in the histogram were mean ± SD of three independent experiments. **c** CD3^+^ T cells or activated CD3^+^ T cells were cultured with MMC-treated MSCs, Ob-MSCs, De-MSCs and Re-MSCs in 96-well plates for 72 h. The proliferation of T cells was assayed by tritiated thymidine ([^3^H]TdR) incorporation. Values of cpm presented were mean ± SD. *T* T cells, *aT* T cells activated by anti-CD3 and anti-CD28, *aT + M* activated T cells co-cultured with MSCs, *aT + Ob* activated T cells co-cultured with Ob-MSCs, *aT + De-M* activated T cells co-cultured with De-MSCs*, aT + Re-ob* activated T cells co-cultured with Re-MSCs (*n* = 3/group). ^*^ *P* < 0.05, ^**^ *P* < 0.01. One-way ANOVA test was employed for intergroup comparison

After we characterized the osteogenic potential of De-MSCs, we further biologically explored the immunogenicity during the osteogenic differentiation. We first assessed the expression of co-stimulatory molecules on MSCs, De-MSCs, Ob-MSCs, and Re-MSCs. The data revealed that MSCs and De-MSCs did not express CD80, CD83, CD86, HLR-DR, and MHC-ABC, which regulate positive immune response. Meanwhile, both of the populations (MSCs and De-MSCs) highly expressed PD-L1 and B7-H3, which are involved in negative immune response at most, while PD-L2 not. Notably, with the differentiation, Ob-MSCs and Re-MSCs increased the expression of CD80, CD83, CD86, and HLA-DR and decreased the expression of PD-L1 and B7-H3, compared to their counterpart MSCs and De-MSCs. Moreover, Re-MSCs exhibited statistically higher expression of CD80, CD86, lower expression of PD-L1, B7-H3 than Ob-MSCs did (*P* < 0.05, respectively, Fig. 4a, b).

It had been reported that MSCs could suppress the immune response of PBMCs stimulated by alloantigens or mitogens, including phytohemagglutinin and concavalin A (Con A) [22]. Here, to study the immunological influence of MSCs, De-MSCs, Ob-MSCs, and Re-MSCs, we stimulated human T cells with anti-human CD3 and CD28 antibodies, and incubated with MSCs, De-MSCs, Ob-MSCs, and Re-MSCs pretreated by MMC, respectively. As shown in Fig. 4c, both undifferentiated and differentiated cells could remarkably inhibit T cell proliferation (compared to activated T cells, *P* < 0.05, respectively). But differentiated cells had significantly decreased their suppressive effect on T cell proliferation compared with their undifferentiated counterparts (*P* < 0.05, respectively). There was no significant difference of suppressive effect between Ob-MSCs and Re-MSCs groups. Here we proposed that the immunogenicity of MSCs and De-MSCs enhanced during differentiation because of the ascended expression of positively regulated co-stimulatory molecules and descended expression of negatively regulated co-stimulatory molecules.

**Fig. 5:**
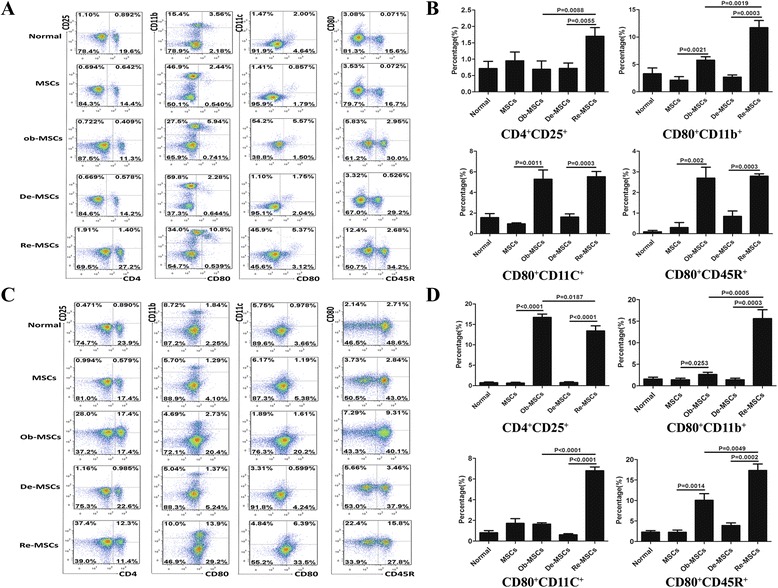
Subpopulations of PBMCs and spleen cells in BALB/c mice immunized by MSCs and De-MSCs. BALB/c mice were injected with MMC-treated MSCs, Ob-MSCs, De-MSCs or Re-MSCs subcutaneously. PBMCs (5 d after injection) and splenocytes (7 d after injection) were collected and analyzed by FCM. CD4^+^CD25^+^ cells, CD80^+^CD11b^+^ cells, CD80^+^CD11c^+^ cells, and CD80^+^CD45R^+^ cells were gated as activated T cells, activated monocytes, activated dendritic cells and activated B cells respectively. (**a**–**b**) Subpopulations of PBMCs. (**c**–**d**) Subpopulations of splenocytes. Values of gated cells presented in the histogram were mean ± SD of three independent experiments. *De-MSCs* dedifferentiated mesenchymal stem cells, *MSCs* mesenchymal stem cells, *Ob-MSCs* MSC-derived osteoblasts, *Re-MSCs* osteoblasts derived from De-MSCs

In the activation of immune cells in vivo, the expression of CD80, a crucial co-stimulatory for initiating immune response, on different populations of PBMCs and splenocytes in immunized mice was analyzed. CD11b^+^ cells, CD11c^+^ cells, CD4^+^ cells, and CD45R^+^ cells were gated as monocytes, DCs, T cells, and B cells, respectively. As we expected, 7 days after immunization, the expression of CD80 on different cell populations from mice immunized with MSCs or De-MSCs, showed a similar profile to that from the mice treated with vehicle. But, the number of CD80^+^CD11b^+^, CD80^+^CD11c^+^, and CD80^+^CD45R^+^ cells increased in PBMCs from the mice immunized with Ob-MSCs and Re-MSCs than with MSCs and De-MSCs (*P* < 0.05, respectively, Fig. 5a, b). The number of activated T cells (CD4^+^CD25^+^) also increased in differentiated cell-primed groups. Meanwhile, the number of CD4^+^CD25^+^ and CD80^+^CD11b^+^ cells was significantly more in De-MSC-primed group than in MSC-primed group. Interestingly, this primed action was observed more obviously in the spleen cells (Fig. 5c, d). Thus, at the current time, these results were consistent with the notion that MSCs and De-MSCs had low immunogenicity [22], while their immunogenicity enhanced during their differentiation.

## Discussion

MSCs are indispensable in regenerative medicine, specifically in bone tissue engineering. However, MSCs derived from different tissues display undesirable therapeutic effects in various preclinical studies because of low survival and differentiation potential as well as unexpected immunogenicity in vivo [9, 13]. In the present study, we isolated MSCs from human placenta and developed a cell population termed De-MSCs via induced osteogenic differentiation and dedifferentiation [15, 17]. We demonstrated that De-MSCs can regain their multilineage differentiation into osteoblasts, adipocytes, and chondrocytes [23], being morphologically and phenotypically similar to uncommitted MSCs. Therefore, we further explored the osteogenic ability of MSCs and De-MSCs both in vitro and in vivo. Compared to MSCs, De-MSCs exhibited a predisposition to the osteoblastic lineage supported by increased osteogenic gene expression and enhanced ALP production.

It is widely accepted that BMP2 plays an important role not only in adjusting the proliferation and differentiation of stem cells [24], but also in osteogenic differentiation and bone formation [25]. As a major transcription factor, Runx2 is essential in bone development and more effectively when introduced together with BMP2 [26, 27]. Lying downstream of Runx2, Osx directs the differentiation of pre-osteoblasts into mature osteoblasts [26, 28]. Meanwhile, as a result of the early gene introduction of BMP2, ALP activity and matrix mineralization are promoted [29–31]. In our in vitro study, De-MSCs exhibited higher potential of proliferation and differentiation than MSCs, illustrated by the proliferation curves of MSCs and De-MSCs during their osteogenic differentiation. Upon osteo-induction, Re-MSCs exhibited statistically higher level of BMP2, Runx2 and Osx mRNA than Ob-MSCs did, which paralleled a similar induction of ALP activity. In vivo, we implanted collagen scaffolds loaded with MSCs and De-MSCs in SCID mice. Similar to the observation in vitro, the ALP activity of implanted De-MSCs and the mRNA levels of BMP2, Runx2 and Osx in implanted De-MSCs were remarkably higher than those of implanted MSCs. The immunohistochemistry staining of collagen II and H&E further evidenced morphologically that De-MSCs had higher osteogenic potential compared to MSCs in situ. These data suggest that De-MSCs have osteogenic superiority to MSCs and may function as a potential cell candidate for bone regeneration.

To date, MSCs have shown great potential in clinical applications, such as for bone regeneration, cardiac repair and treatment of liver diseases [32–35]. However, the engraftment efficiency, migration behaviors, and functionality of transplanted MSCs in a living animal model are poorly understood. Rui et al showed that De-MSCs could survive longer in unfavorable environment, the mechanism of which is associated with microRNA3, resulting in epigenetic memory gained by priming with osteogenic induction medium [17].

MSCs express immunosuppressive molecules and various growth factors which can facilitate tissue repair and maintain immune homeostasis [36]. MSCs have no or low expression of co-stimulatory molecules CD40, CD80, CD86, moderate expression of MHC class I molecules, absent expression of MHC class II molecules, which contribute to low immunogenicity of MSCs [37]. In order to better understand the immunogenic properties of De-MSCs, we compared the immunogenicity and co-stimulatory expression of MSCs and De-MSCs during their osteogenesis. It is well-documented that co-stimulatory molecules play an essential role in modulating immune response through a variety of mechanisms [38]. Specifically, the B7 family members provide a key checkpoint in the regulation of T cell immunity. The upregulation of the expression of CD80 (B7-1) and CD86 (B7-2) on antigen-presenting cells can directly influence the activation and proliferation of T cells, initiating immune response [39]. In contrast, PD-1/PD-L1 pathway attributes to damping T cell responses, promoting T cell tolerance and preventing autoimmunity at most [18]. Among the immunoglobulin superfamily, CD83 (HB15) is necessary for effective DC-mediated activation of naive T cells, thymic T cell maturation and the regulation of B cell activation and homeostasis [40]. In the present study, we explored the expression of B7 family members of the immunoglobulin superfamily proteins on different cell populations. As stated, MSCs, as well as De-MSCs, did not express co-stimulatory molecules CD80, CD83, CD86, but highly expressed co-inhibitory molecules PD-L1 and B7-H3. But Ob-MSCs and Re-MSCs expressed higher CD80, CD83, and CD86, lower PD-L1 and B7-H3 than MSCs and De-MSCs did. In addition, MSCs and De-MSCs significantly suppressed T cell proliferation more strongly than Ob-MSCs and Re-MSCs did. Thus, Ob-MSCs and Re-MSCs gained higher immunogenicity upon osteogenic induction than MSCs and De-MSCs in vitro. However, the cell population pattern primed with MSCs or De-MSCs was similar to the one with vehicle delivery. In vivo study, we showed that activated T cells, B cells, monocytes, and macrophages were found in PBMCs and splenocytes of mice immunized with MMC-treated Ob-MSCs and Re-MSCs. Furthermore, the activation of Re-MSCs was higher than Ob-MSCs. These results indicate that the immunogenicity of differentiated MSCs and De-MSCs increase functionally compared with the undifferentiated counterparts, and Re-MSCs elicit more enhanced immunogenicity compared to Ob-MSCs. Given the complexity of immune-modulation in vivo, it is plausible that other partner cell types are involved in the immune-regulated effect of MSCs. During MSCs differentiation, multiple mechanisms are involved in the fate determination process, including genetic and epigenetic regulation [17]. B7-H3 is expressed on antigen-presenting cells and downregulates T cell functions by engaging an unknown counter-receptor on T cells. As well, it is also identified to have a role in the bone-immune interface, playing a positive regulatory role in bone formation [41, 42]. We demonstrated that undifferentiated cells had upregulated immunogenicity, in association with differentiation. Consequently, we speculate that the mechanism of this phenomenon may be related to both epigenetics and signal pathways. In-depth research needs to be conducted to verify all these.

In summary, De-MSCs share similar morphology, cell surface markers, and lower immunogenicity to MSCs. Moreover, they have higher potential for proliferation and differentiation compared to MSCs during osteogenesis. Our study further supports the notion that De-MSCs may serve as an alternative source of cells with enhancing therapeutic efficacy for regenerative medicine and tissue engineering. However, we have demonstrated that De-MSCs had upregulated immunogenicity as MSCs during their osteogenesis. Thus, the immunologic intervention may serve as a beneficial strategy in MSC-based therapy to maximize the potential of MSCs and De-MSCs. Furthermore, more explicit and detailed mechanisms involved in the differentiation potential and immunogenicity of MSCs and De-MSCs need to be elucidated in the future.

## Conclusions

In conclusion, this study has characterized a cohort of dedifferentiation-reprogrammed stem cells which had prominent survival and differentiation with improved therapeutic potential. De-MSCs had a higher osteogenesis compared to MSCs. We should fully consider the upregulated immunogenicity of De-MSCs with redifferentiation in clinical applications.

## Abbreviations

ALP: Alkaline phosphatase
B7H3: B7-homolog 3
BMP2: Bone morphogenetic protein 2
CCK-8: Cell Counting Kit-8
cDNA: Complementary DNA
Con A: Concavalin A
DC: Dendritic cells
De-MSCs: Dedifferentiated mesenchymal stem cells
DMEM-LG: Dulbecco’s modified Eagle’s medium-low glucose
FBS: Fetal bovine serum
FCM: Flow cytometry
GAPDH: Glyceraldehyde-3-phosphate dehydrogenase
H&E: Hematoxylin and eosin
IBMX: 3-isobutyl-1-methylxanthine
MHC: Major histocompatibility complex
MLR: Mixed lymphocyte reaction
MMC: Mitomycin C
MSCs: Mesenchymal stem cells
NK: Natural killer cells
NOD/SCID: Nonobese diabetic/severe combined immunodeficient
Ob-MSCs: MSC-derived osteoblasts
OD: Optical density
Osx: Osterix
PBMCs: Peripheral blood mononuclear cells
PD-L1: Programmed cell death 1 ligand 1
PD-L2: Programmed cell death 2 ligand 1
PHA: Phytohemagglutinin
qRT-PCR: Quantitative real-time PCR assays
Re-MSCs: Osteoblasts derived from De-MSCs
Runx2: Runt-related transcription factor 2
TGF-β1: Transforming growth factor beta 1
